# Associations between Teacher- and Student-directed Sexual and Physical Violence in Physical Education

**DOI:** 10.1177/0886260520959640

**Published:** 2020-09-18

**Authors:** Arto Gråstén, Marja Kokkonen

**Affiliations:** 1 Faculty of Sport and Health Sciences, University of Jyväskylä, Jyväskylä, Finland; 2 School of Education, University of Tasmania, Launceston, Tasmania, Australia

**Keywords:** school, interpersonal, verbal, non-verbal, harassment, victimization

## Abstract

This study examined relationships between teachers’ perceptions of verbal and non-verbal sexual harassment and physical violence against teachers and among students in physical education (PE). Participants were 175 (females 122, males 53) Finnish PE teachers between 27 and 62 years (*M* = 44.8 ± 9.2 years). The cross-sectional data were collected by an anonymous online survey in the fall semester 2018. The findings showed that (a) higher levels of verbal sexual harassment and physical violence among students were associated with higher levels of equivalent types of violence against PE teachers, (b) higher levels of verbal and non-verbal sexual harassment among students were associated with higher levels of physical assaults among students, whereas only non-verbal sexual harassment was associated with physical violence against teachers, and (c) verbal sexual harassment and physical violence among students occurred more frequently in PE classes instructed by less experienced teachers. The results indicated that to prevent both teacher- and student-directed verbal sexual harassment and physical violence in school PE, special attention could be given to the positive development of student–student relationships.

## Introduction

School violence is a growing concern in many Western countries (Maeng et al, 2020). The risk for being sexually harassed or physically assaulted may be higher for physical education (PE) teachers than those in other school subjects, as non-existent desk-time does not restrict social opportunities (Garn et al., 2011) and movements or injury prevention in sport classes are typically performed through physical contact or touching (Öhman & Grunberg-Sandell, 2015). Although violent behavior between students in schools has been widely documented (Lenzi et al., 2014; Moon & Alarid, 2015; Moon & Jang, 2014; Poling et al., 2019; Yablon, 2017) and research on teachers’ experiences of different types of interpersonal violence has started to appear (Bounds & Jenkins, 2018; Kauppi & Pörhölä, 2012; Longobardi et al., 2019; Moon & McCluskey, 2018), the relation between teacher- and student-directed violent behavior in schools, especially in PE classes, remains unclear. The primary objective of this study on sexual and physical violence both by students against teachers and between students themselves was to shed light on these associations in a school subject (PE) that is very different in nature from other subjects on the curriculum. 

Interpersonal violence includes both sexual and physical violence. Sexual harassment as a form of sexual violence (Ferrara et al., 2019; Krug et al., 2002) is defined as unwanted, threatening or offensive touching (physical), gesturing (non-verbal) and inappropriate jokes or offensive, sexually charged comments (verbal) (Bendixen et al., 2018; Fasting, 2015). Non-verbal sexual harassment includes sexually colored facial or bodily signals, such as whistling, winking, leering, howling, or kissing sounds (Bendixen et al., 2018). In turn, verbal sexual harassment is manifested by derogatory sexual remarks, sexual jokes, sexual questions, requests for intercourse, and the spreading of sexually charged rumors (Bendixen et al., 2018; Chiodo et al., 2009). The prevalence of sexual harassment in Nordic schools appears to be less common than in some other countries such as the United States (Kauppi & Pörhölä, 2012; Kurtz et al., 2018). For instance, in a study of U.S. students in grades 9 to 12 (51% girls), about 65% of girls and 35% of boys reported experiencing some type of sexual harassment at least once during the past school year (Crowley et al., 2019). In contrast, in the national School Health Promotion Study conducted in Finland 4.5% of students in grades 8 and 9 (51% girls) and 3.5% of boys had experienced some type of sexual harassment at school during the past 12 months (Finnish Institute for Health and Welfare, 2019). Worldwide, only a few studies on teacher-perceived sexual harassment perpetrated by their students have been published. For example, in a Finnish survey conducted by the Institute of Criminology and Legal Policy (Salmi & Kivivuori, 2009), approximately 3% of Finnish school teachers reported harassment by students, whereas only 1% of K-12 teachers in the U.S. reported that sexual harassment in their workplace is very or extremely common (Kurtz et al., 2018). Based on a nationwide web-based survey in Dutch secondary schools, nearly 3% of teachers had experienced some type of sexual harassment (verbal, non-verbal, or physical) by their students (Mooij, 2011). As these examples show, the variation in the research questions, samples and measures used makes comparison between studies very problematic. Given the paucity of quantitative data on sexual harassment in schools (Kauppi & Pörhölä, 2012), there is a need to examine the phenomenon both against teachers and among students in addition to physical assaults generally.

Physical violence is described as the intentional, one-time or repeated rough infliction of pain or bodily injuries by another person that involves a potential risk for visible or non-visible physical harm (Krug et al., 2002). Previous studies of physical incidents in schools indicate that the prevalence of physical violence varies widely between countries. For example, in a recent Finnish national study, nearly 14% of girls and 20% of boys aged 14–16 reported experiencing physical threats at school during the past year (Finnish Institute for Health and Welfare, 2019). By contrast, about 3% of male students and 2% of female students aged 12–18 in the U.S. reported being victimized at school when victimization was considered as more serious than bullying but less serious than non-fatal violent assaults (Musu et al., 2019). Regarding violence against teachers, about 26% of Finnish elementary and secondary school teachers reported occasionally being subjected to verbal, non-verbal, and physical assaults by students (Kauppi & Pörhölä, 2012). In a national study conducted in the US, 44% of K-12 teachers reported at least one experience of physical violence during the current or past school year (McMahon et al., 2014). In a Slovakian study (Dzuka & Dalbert, 2007), 5% of secondary school teachers had experienced physical violence by students within the past thirty days. It is clear from the previous studies that the total prevalence of physical violence among students and against teachers varies widely across countries. Although school violence may have negative impacts on the whole school community (Dzuka & Dalbert, 2007; James et al., 2008; Kauppi & Pörhölä, 2012; Moon & McCluskey, 2018), the relationships between teacher- and student-directed violence remain, owing in part to inadequate documentation, largely unknown.

A review of previous studies published in the field showed that past research has mostly focused on school violence among students (Bounds & Jenkins, 2018; Gerberich et al., 2014; Mármol et al., 2018), whereas violence against teachers, especially sexual harassment, has remained largely undocumented (Kurtz et al., 2018; Longobardi et al., 2019). Furthermore, past studies have typically included only one or a few types of violence (Buda, 2009; Lester et al., 2017; Mooij, 2011), although school violence is considered as a multilevel phenomenon (Frey et al., 2010). Given that violence against teachers has previously been explained by teacher gender (Mooij, 2011; Salmi & Kivivuori, 2009), length of teaching experience (Lokmić et al., 2013), and student age (Chen & Astor, 2009; Dzuka & Dalbert, 2007), these covariables were included in the present analysis. This study aimed to contribute to filling the present research gaps by investigating both sexual and physical violence by students against teachers and between students themselves in school PE classes, topics that have not yet been addressed in the same study.

**Figure 1.**
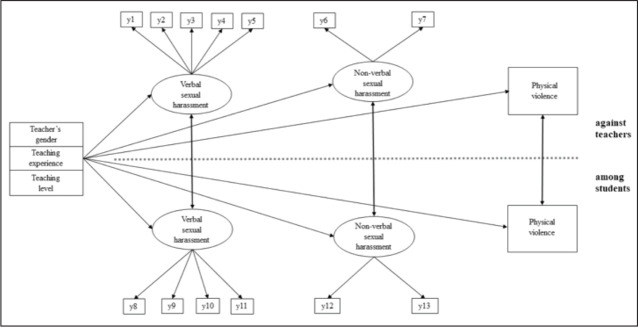
The theorized model including covariables, verbal sexual harassment, non-verbal sexual harassment, and physical violence against teachers and among students in PE classes (y1–y13 = observed variables). For the sake of clarity, associations between verbal, non-verbal sexual harassment, and physical violence are not illustrated.

Specifically, the aims of this study were to examine (a) the relationships between teacher- and student-directed physical violence and non-verbal and verbal sexual harassment perceived by PE teachers, and (b) the relationships between antecedents (teacher’s gender, teaching level, and years of teaching experience) of teacher- and student-directed physical violence and non-verbal and verbal sexual harassment (Figure 1). Based on previously established associations, physical violence and non-verbal and verbal sexual harassment among students were expected to be positively associated with higher levels of sexual harassment and physical violence against teachers. Female PE teachers, middle school PE teachers, and PE teachers with less teaching experience were expected to report higher sexual harassment and physical violence against teachers than their counterparts.

## Methods

### Participants

Participants were 175 PE teachers between the ages of 27 and 62 years (*M* = 44.8 ± 9.2 years) recruited across eighteen regions in Finland (Table 1). About 96% of participants reported themselves as heterosexual, nearly 2% as mostly heterosexual, and 2% as homosexual or other. Mean teaching experience was 16 years, ranging from 1 to 38.

**Table 1.** Participant Demographics (N = 175).

	Quantity	Percentage	Verbal Sexual Harassment against Teachers	Verbal Sexual Harassment among Students	Non-verbal Sexual Harassment against Teachers	Non-verbal Sexual Harassment among Students	Physical Violence against Teachers	Physical Violence among Students
Gender								
Female Male	12 253	69.7 30.3	1.25 (.27) 1.26 (.28)	1.64 (.58) 1.69 (.57)	1.10 (.26) 1.14 (.35)	1.61 (.74) 1.60 (.75)	1.04 (.20) 1.02 (.14)	2.46 (1.10) 2.36 (1.08)
Region								
Uusimaa Pirkanmaa Southwest Finland Central Finland North Ostrobothnia Other	52 18 17 16 11 61	29.9 10.3 9.8 9.2 6.3 34.5	1.31 (.32) 1.27 (.34) 1.22 (.23) 1.17 (.24) 1.27 (.31) 1.25 (.23)	1.73 (.55) 1.59 (.55) 1.64 (.69) 1.40 (.42) 1.55 (.47) 1.74 (.65)	1.10 (.24) 1.14 (.41) 1.09 (.26) 1.07 (.26) 1.23 (.41) 1.12 (.28)	1.63 (.64) 1.42 (.52) 1.88 (1.15) 1.30 (.49) 1.68 (.56) 1.65 (.83)	1.08 (.27) 1.06 (.24) 1.00 (.00) 1.00 (.00) 1.00 (.00) 1.03 (.18)	2.40 (1.07) 2.50 (1.15) 3.12 (1.05) 1.94 (.68) 2.36 (1.21) 2.43 (1.13)
Teaching level								
Elementary 1–6 Middle school 7–9 High school Vocational school Higher education Elementary + Middle school 1–9 Middle school 7–9 + High school	9 64 25 4 2 15 56	5.1 36.6 14.3 2.3 1.1 8.6 32.0	1.21 (.26) 1.29 (.27) 1.16 (.32) 1.29 (.17) 1.00 (.00) 1.41 (.37) 1.25 (.25)	1.40 (.45) 1.76 (.56) 1.42 (.50) 1.71 (.48) 1.00 (.00) 1.79 (.56) 1.72 (.61)	1.11 (.33) 1.13 (.32) 1.00 (.00) 1.30 (.45) 1.00 (.00) 1.27 (.42) 1.09 (.24)	1.50 (.71) 1.67 (.76) 1.36 (.62) 1.20 (.27) 1.00 (.00) 1.63 (.55) 1.74 (.80)	1.22 (.44) 1.03 (.18) 1.00 (.00) 1.00 (.00) 1.00 (.00) 1.07 (.26) 1.02 (.14)	2.56 (.88) 2.66 (.98) 1.48 (.51) 2.00 (.00) 1.00 (.00) 3.00 (1.00) 2.52 (1.17)
Teaching experience								
1–5 years 6–10 years 11–15 years 15 years	30 27 38 80	17.0 15.0 22.0 46.0	1.33 (.24) 1.30 (.40) 1.24 (.27) 1.23 (.25)	1.82 (.60) 1.69 (.66) 1.60 (.63) 1.63 (.54)	1.20 (.34) 1.12 (.29) 1.11 (.31) 1.08 (.25)	1.73 (.88) 1.65 (.88) 1.59 (.81) 1.57 (.63)	1.07 (.25) 1.04 (.20) 1.08 (.27) 1.01 (.11)	2.80 (1.06) 2.59 (1.34) 2.50 (1.16) 2.21 (.95)

*Note.* Means and standard deviations (in the parentheses) for each group are presented in vertical columns.

Nearly 15% of the target population (approx. 1,200 PE teachers) participated in the study (Association of Physical and Health Educators in Finland, 2019). Setting statistical significance level at .05 and power at 85% and 90%, the estimated sample size was 177 and 223 subjects. This indicated that the number of participants in the current research tasks was at the acceptable level. All participants who completed the online questionnaire with demographic information were included in the study (convenience sampling).

### Study Design

The cross-sectional data were collected anonymously by a web survey in September–November 2018. The link to the online survey was published on the website of the Association of Physical and Health Educators in Finland. It was also emailed to individual school principals and PE teachers. On the opening page of the online survey, prospective respondents were informed about the objectives and methodology of the study, potential disadvantages of participation, data processing methods, channels of publication, and the voluntary and anonymous nature of participation. To continue from the opening page, participants had to give their informed consent by ticking a box. The survey data were automatically saved on the local university database and processed by the researchers. The study was approved by the local university ethics committee.

## Measures

Using a structured response form, PE teachers answered questions about their gender, present level of teaching, and length of teaching experience. Present teaching level comprised seven options (elementary 1–6, middle school 7–9, high school, vocational school, university or university of applied sciences, combined elementary and middle school 1–9, and combined middle and high school). Teaching experience was measured with the direct question “In total, how many years you have worked as a PE teacher during your work career?” and the responses were rounded to the nearest year.

Sexual (verbal and non-verbal sexual harassment) and physical violence against PE teachers and among students in the school PE context were assessed using the scales modified from previous harassment studies in the school context. The item stem was “During the last school year, how often have the students in your groups….” Verbal sexual harassment against PE teachers was measured using a latent variable comprising items (y1–y5) used in previous studies (Dahlqvist et al., 2016; Peter et al., 2016; Witkowska & Kjällberg, 2005): “Spread sexually colored rumors about you,” “Called you names or insulted you in a degrading, sexually colored way,” “Called you a slut,” “Asked about your gender or your sexual orientation in an inappropriate context,” and “Commented on your gender or your sexual orientation in an offensive way.” Verbal sexual harassment among students was measured using a latent variable comprising the following items (y8–y11): “Spread sexually colored rumors about some student,” “Called names or insulted some student in a degrading, sexually colored way,” “Called some student a slut,” and “Called some student a homosexual.” PE teachers rated the frequency of each incident over the past year on a Likert scale from *never* (1) to *almost daily* (5).

Non-verbal sexual harassment against PE teachers was measured using a latent variable constructed from the following two items (y6–y7) modified from the Sexual Experiences Questionnaire (SEQ—DoD; Fitzgerald et al., 1999): “Gestured or signaled to you in a sexually charged or suggestive way (e.g., hand signs, body language)” and “Made sounds that you perceived as inappropriate or sexually colored (e.g., shouted after you, whistled, gasped or smacked).” Similarly, non-verbal sexual harassment among students was measured using a latent variable comprising the items (y12–y13): “Gestured or signaled to some student in a sexually charged or suggestive way (e.g., hand signs, body language)” and “Made sounds that some student perceived as inappropriate or sexually colored (e.g., shouted after some student, whistled, gasped or smacked).” Both subscales were measured on five-point scales from *never* (1) to *almost daily* (5).

Physical violence against PE teachers and among students was assessed using a single item in each case. For physical violence against teachers, it was “Pushed, bumped, slapped, pinched, and punched or otherwise physically assaulted you” and for physical violence among students it was “Pushed, bumped, slapped, pinched, and punched or otherwise physically assaulted other students.” Both items were rated using a five-point Likert scale from *never* (1) to *almost daily* (5).

### Data Analyses

Normality of the distribution, outliers, and missing values were examined. Correlation coefficients, means, and standard deviations were calculated for each study variable. To test the relationships between the covariables (teacher’s gender, teaching level, teaching experience), non-verbal and verbal sexual harassment, and physical violence against teachers and among students, a path model was implemented.

The chi-square test (*χ^2^*) was used to test the overall fit of the model. A non-significant difference (*p* > .05) between the observed distribution and the theoretical distribution demonstrates acceptable fit of the data. To examine the goodness of fit of the model, the standardized root mean square residual (SRMR), the root mean square error of approximation (RMSEA), the comparative fit index (CFI), and the Tucker–Lewis index (TLI) were used. A SRMR value of less than .06 is considered as a good model fit and a RMSEA value of .08 or less indicates an acceptable model fit. For the CFI and TLI indices, values greater than .95 are indicative of an excellent model fit (Kline, 2005). The proportion of variance was analyzed using a squared multiple correlation value (*R*^2^). The preliminary analyses, including missing value analysis and descriptive statistics, were performed using SPSS 26.0 and the modelling with Mplus 8.3.

## Results

### Preliminary Analyses

The data were approximately normally distributed. Based on the standardized values (± 3.00), three significant outliers were found in the scores for non-verbal sexual harassment against PE teachers (Tabachnick & Fidell, 2007). These outliers caused an unacceptable Cronbach’s alpha value (*α* = .49) and were removed. The alphas then supported the internal consistency of the subscales of non-verbal (*α* = .69) and verbal sexual harassment (*α* = .82) against teachers and non-verbal (*α* = .76) and verbal sexual harassment (*α* = .83) among students. The percentage of missing values was 0.5% (13 out of 2,655 values), as 10 teachers did not fully complete the online form. The missing completely at random (MCAR) test (*χ^2^* = 28.29, *df* = 22, *p* = .166) indicated that the data with and without missing values were similar. Therefore, the missing values were assumed to be MCAR (Little & Rubin, 2002).

### Descriptive Statistics

Correlation coefficients, means, standard deviations, and the composite reliability of the study variables were analyzed (Table 2). The correlations between the study variables ranged from weak positive to moderate positive. The strongest positive correlation was found between verbal and non-verbal sexual harassment among students. The mean scores for non-verbal and verbal sexual harassment and physical violence against PE teachers were relatively low when compared the scores among students. Nearly 4% of PE teachers reported having experienced physical violence and 5.2% non-verbal sexual harassment by their students a few times per year, and 0.6% reported experiencing verbal sexual harassment monthly. About 5% of PE teachers reported witnessing physical violence among students almost daily, and nearly 5% non-verbal sexual harassment and 8% verbal sexual harassment among students weekly or almost daily.

**Table 2.** Correlations, Means (*M*), Standard Deviations (*SD*), and Composite Reliability (CR) of the Study Variables.

	1	2	3	4	5	6	*M*	*SD*	CR
Against teacher									
1 Non-verbal sexual harassment	–	–	–	–	–	–	1.09	0.25	0.75
2 Verbal sexual harassment	0.31***	–	–	–	–	–	1.09	0.28	0.83
3 Physical violence	0.23**	0.40***	–	–	–	–	1.04	0.20	–
Among students									
4 Non-verbal sexual harassment	0.24**	0.38***	0.36***	–	–	–	1.62	0.75	0.75
5 Verbal sexual harassment	0.32***	0.45***	0.35***	0.68***	–	–	2.01	0.79	0.83
6 Physical violence	0.22**	0.28***	0.30***	0.46***	0.62***	–	2.43	1.10	–

*Note*. ***p < .001. **p < .01.

### The Model of Sexual and Physical Violence

**Figure 2.**
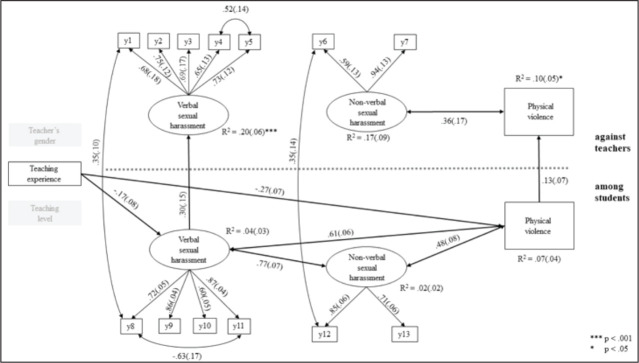
Standardized results of path analysis for verbal sexual harassment, non-verbal sexual harassment, and physical violence against teachers and among students in PE classes (y1–y13 = observed variables). All paths are significant at *p* < .05 level.

To test the relationships between PE teacher’s gender, teaching level, teaching experience, non-verbal and verbal sexual harassment, and physical violence against PE teachers and among students, a path model was implemented. The theorized model showed unacceptable model fit for the data (*χ^2^*(104) = 206.63, *p* < .001, CFI = .85*, TLI* = .78, RMSEA = .077, 90% CI [.06, .09], SRMR = .064). Based on the modification indices, the model with one-way arrows (from verbal sexual harassment and physical violence among students to the equivalent variables against PE teachers) and the correlations between the residuals of the items y1 (Spread sexually colored rumors about you) and y8 (Spread sexually colored rumors about some student*)* in addition to the residuals of y6 (Gestured or signaled to you in a sexually charged or suggestive way*)* and y12 (Gestured or signaled to some student in a sexually charged or suggestive way) showed acceptable fit to the data (*χ^2^*(100) = 141.49, *p* = .004, CFI = .94, TLI = .91, RMSEA = .050, 90% CI [.03, .07], SRMR = .055) (Figure 2).

Direct paths were found from verbal sexual harassment among students to verbal sexual harassment against PE teachers and from physical violence among students to physical violence against PE teachers, indicating that frequent verbal sexual harassment and physical violence among students were reflected in higher levels of verbal sexual harassment and violence against PE teachers. Verbal and non-verbal sexual harassment and physical violence among students correlated with each other, and a higher frequency of sexual violence was linked to a higher frequency of physical assaults, whereas only non-verbal sexual harassment and physical violence against PE teachers were associated with each other. Lower length of teaching experience was associated with higher levels of verbal sexual harassment and physical violence among students, showing that verbal harassment and physical incidents occurred more often in PE classes instructed by less experienced teachers. Squared multiple correlations (*R*^2^) showed that the model explained 20% of the variability of verbal sexual harassment against PE teachers and 10% of the variability of physical violence against PE teachers.

## Discussion

The present study examined the relationships between verbal and non-verbal sexual harassment and physical violence against PE teachers and among students in school PE classes. It extends previous studies in the field by investigating concurrently teacher- and student directed violence, which has not yet been empirically tested in the PE domain. The main findings showed that (a) higher levels of verbal sexual harassment and physical violence among students were reflected in higher levels of equivalent types of violence against PE teachers, (b) higher levels of verbal and non-verbal sexual harassment were linked to higher physical assaults among students, whereas only non-verbal sexual harassment was associated with physical violence against PE teachers, and (c) verbal sexual harassment and physical incidents among students occurred more frequently in PE classes instructed by less experienced PE teachers.

There are some limitations in the present study, which should be considered when interpreting the results. First, while the cross-sectional findings provide a useful picture of the relationships between the types of interpersonal violence that occur in PE classes, they do not reveal causality. Second, the data on student–student violence were not collected from students, which could reveal violence among students hidden from teachers and authorities. Third, the study was implemented in Finland, where social relationships between students and teachers are rather informal and equal in schools (Heo et al., 2017), while school culture is somewhat authoritative compared with other Nordic countries (Yoon & Järvinen, 2016). This means that while teachers are easily approachable by students, the threshold for undesirable student behavior may also be lower. Therefore, the results should be interpreted with caution, as they may not be applicable in some other school cultures. Finally, the study could have benefitted from a larger sample of PE teachers across the country, as it was impossible to know whether the sample was representative of the PE teachers who have most frequently been subjected to sexual or physical violence by their students. This is often the case when using anonymous data collection procedures, as the researchers must choose between a greater and a lesser evil, especially in the case of sensitive or undesirable behaviors.

Mindful of these considerations, the present findings could be interpret through the social-ecological model (Bronfenbrenner, 1977), which comprises individual characteristics of teachers (i.e., gender, length of teaching experience) and their interactions with the social context (i.e., school PE classes) (Espelage, 2014). For example, a social-ecological model based KiVa-program (Ahtola et al., 2012; Salmivalli et al., 2010a, 2010b, 2011) proposed that face-to-face discussions with victims and bullies were effective methods to prevent bullying among students and against teachers in schools. Specifically, the KiVa-program includes student lessons and themes with discussions, video films, and exercises done in dyads and small groups, a virtual learning environment (an antibullying computer game for primary school students, an Internet forum “KiVa Street” for secondary school students), a parents’ guide and symbols (posters, highly visible vests for teachers supervising recess time), reminding both students and school personnel of the program. The lesson topics cover a variety of issues related to group interaction and group pressure, the mechanisms and consequences of bullying, different forms of bullying, and especially, what the students can do together in order to counteract bullying and support the victimized peers. It must be noted that the KiVa actions were mostly delivered in classroom settings, while this study investigated the outcomes in PE classes.

Applying the present results into practice, a prevention of teacher-directed violence in PE could be achieved in the first instance by tackling student–student violence. Promoting a positive school climate (Cornell et al., 2016; Salmivalli et al., 2011; Smith et al., 2014) and developing students’ social and emotional competence skills, such as conflict resolution (Keane, 2011) and cooperation (Cain, 2011) have been proposed as potentially effective strategies for decreasing the incidence of violence among students (as described in the KiVa program). In more practical terms, PE teachers could, for instance, create opportunities for students to participate in leadership and decision-making roles, use teaching strategies that increase social learning (e.g., conflict resolution and cooperative learning) and collaborate with their counterparts to address students’ behavior (Piscatelli & Lee, 2011).

Furthermore, to support the positive development of social and emotional competence, specifically in school PE, students should be provided with a well-structured environment that gives clear guidelines (open teacher–student communication) and opportunities for social learning (cooperation between students), provides optimal challenges, and offers detailed feedback on how to achieve desired outcomes (Taylor & Ntoumanis, 2007). Past studies have also revealed that school violence is much more likely to occur when students feel that grades are impossible to achieve (Martin, 2011) or perceive the school curriculum to be irrelevant (Ornstein et al., 2012). Students also play a critical role in promoting a positive school climate (Piscatelli & Lee, 2011), as associations of larger class sizes and higher total numbers of students with higher levels of violence have also been found (Reeves, 2010). In PE, students could make a conscious effort to be a positive role model for others, hold high expectations for their own behavior and that of others, and demonstrate good sportsmanship within the boundaries set (Smith et al., 2014). Thus, several strategies aimed at promoting a positive school climate in PE classes currently exist.

It should be mentioned that the majority of PE teachers in the present sample were teaching in middle schools, where, following the major gendering practice in Finnish schools, students are typically taught in single-sex groups (Turpeinen et al., 2012). Some studies have indicated that single-sex grouping can increase gender stereotyping (Wong et al., 2018), in turn exacerbating aggressive behaviors in schools (Lee et al., 1994). Rinehart and Espelage (2016) showed, further, that when teachers perceive schools as committed to bullying prevention, students reported less sexual harassment and victimization. This indicates that preventing any kind of student aggression and misbehavior at an early stage would be important in creating a safe and nonviolent PE teaching and learning environment.

Measures to eliminate violence in school PE could include (a) ensuring that PE teachers know how to recognize cases of violence, and to whom to refer cases of violence (e.g., a clear contact protocol for police and other authorities in the case of serious incidents of violence), (b) recognizing the potential contribution of students to the creation of nonviolent classes and providing appropriate structures (e.g., student councils for formal student participation in school management), (c) ensuring that teaching materials and classes promote positive values and tolerance (e.g., students are required to use language in a precise and appropriate manner, with a prohibition on sexual labelling or homophobic slurs), (d) respecting diversity (e.g., groups and teams are regularly changed and do not always consist of the same students), and (e) clearly informing students and parents about how their children can improve their PE performance (Cohen, 2013).

However, the development of a violence-free and positive school climate is not limited to PE classes but concerns the whole school community, starting from a human-centered leadership. All such improvements rely on good cooperative working arrangements between teachers, students, and other school personnel in which the principle of accessibility (e.g., an open-door policy, students not always getting what they want but being heard and respected) supported by clear and consistent communication (e.g., principals know students’ names) is crucial (Smith et al., 2014).

Finally, verbal sexual harassment and physical incidents among students occurred more frequently in PE classes instructed by less experienced teachers. In a study on violence in schools and teacher well-being in a sample of Italian teachers**,** De Cordova et al. (2019) suggested that longer serving teachers may be better at building good relationships with their students, as they can influence their students via their teaching approaches and are less sensitive to provocation by students. Good communication with students, which includes setting boundaries for acceptable behavior (Aldrup et al., 2018), and creating a pleasant working atmosphere (Lokmić et al., 2013) may help less-experienced teachers prevent student misbehavior in PE classes.

It is also possible that longer serving teachers consider verbal sexual harassment as “typical” inappropriate language rather than as sexual harassment. If so, this could explain their lower reporting rate. Kurtz et al. (2018) found that a major problem in estimating the frequency of verbal sexual harassment is that most teachers who experience or witness this do not report it. Therefore, PE teachers could be trained to recognize inappropriate sexual harassment and encouraged to apply lower thresholds for reporting sexual harassment or violent behavior (Maeng et al., 2020), strategies that could be effective in preventing violence in schools. PE teacher education could, for example, question the endorsement of a traditional masculine ideology that has been found to increase adults’ acceptance of homophobic language (Rosenberg et al., 2017). Furthermore, cooperation between families and schools could support positive student–student as well as teacher–student relationships. This in turn could manifest as a lower frequency of sexual harassment and violent incidents among students (Aldrup et al., 2018).

## Conclusions

The key findings of this study that higher levels of verbal sexual harassment and physical violence among students were associated with higher levels of these behaviors directed against teachers support earlier findings that violence against teachers in schools is a multilevel phenomenon. Second, all types of interpersonal violence and verbal and non-verbal sexual harassment were linked to a higher rate of physical assaults among students, indicating that early intervention irrespective of the form of aggressive behavior could act as an effective strategy in promoting violence-free student relationships in PE classes. In line with previous studies suggesting that longer serving PE teachers may be better at building good relationships with their students and less sensitive to student provocation, less experienced teachers reported more frequent occurrence of verbal sexual harassment and physical incidents among students in their PE classes.

Based on these findings, the main focus in preventing undesirable student behavior in PE classes, and in schools generally, could be to promote positive relationships among students. Irrespective of the form of aggressive behavior, early intervention, in the first instance through quality pedagogies, may act as an effective strategy in aggression prevention. Supplemental training could be provided to in-service PE teachers to help them identify the different forms of sexual harassment and encourage them to adopt a lower threshold for reporting sexual harassment or violent student behavior. To do this, up-to-date reporting protocols should be implemented in all schools irrespective of the prevalence of incidents. Finally, to raise awareness of teacher- and student-directed sexual and physical violence in PE, the topic should be considered more widely in future PE teacher education programs. Positive relationships between schools and families would also be helpful in developing a school culture where unwanted student behavior could be tackled as a community.

Future studies could aim to capture violent student behavior in single-sex and mixed-sex groups in PE classes over several measurement points, including student self-reports, enabling comparisons of teachers’ and students’ perceptions of sexual and physical violence. In addition, school-based interventions aimed at promoting a positive school climate and students’ social and emotional competence skills would be of great value, ensuring that schools are safe spaces not only for students but also their teachers (Higham, 2018).

## Author Biographies

**Arto Gråstén,** PhD in Sport Sciences, Master of Education, health and physical education teacher, works as a postdoc researcher in the Faculty of Sport and Health Sciences within University of Jyväskylä, Finland and an Adjunct Lecturer in Health and Physical Education within University of Tasmania, Australia. His research comprises exercise motivation, physical education, and fundamental movement skills, physical activity, and across the school-based physical activity interventions.

**Marja Kokkonen,** PhD in Psychology, MSc in Sport Sciences, works as a senior lecturer in the Faculty of Sport and Health Sciences at the University of Jyväskylä, Finland. She is a principal investigator in a research project PREACT that tackles various forms of discrimination in sport and physical education, funded by the Finnish Ministry of Education and Culture 2018–21. She is an expert member of the Division of Equality, Equity, and Sustainable Development in the National Sports Council in Finland.
